# An Inhibitory Effect of Dryocrassin ABBA on *Staphylococcus aureus* vWbp That Protects Mice From Pneumonia

**DOI:** 10.3389/fmicb.2019.00007

**Published:** 2019-01-23

**Authors:** Bangbang Li, Yingli Jin, Hua Xiang, Dan Mu, Panpan Yang, Xianmei Li, Ling Zhong, Junjie Cao, Dan Xu, Qian Gong, Tiedong Wang, Lin Wang, Dacheng Wang

**Affiliations:** ^1^Department of Pharmacology, College of Basic Medical Sciences, Jilin University, Changchun, China; ^2^College of Animal Science and Technology, Jilin Agricultural University, Changchun, China; ^3^Key Laboratory of Animal Production and Product Quality Safety of Ministry of Education, Jilin Agricultural University, Changchun, China; ^4^College of Animal Sciences, Jilin University, Changchun, China; ^5^Shen Yang Weijia Animal Husbandry Company Limited, Shenyang, China; ^6^College of Humanities & Sciences of Northeast Normal University, Changchun, China; ^7^Key Laboratory of Zoonosis Research, Ministry of Education, Institute of Zoonosis, College of Veterinary Medicine, Jilin University, Changchun, China

**Keywords:** dryocrassin ABBA, pneumonia, direct inhibitor, *Staphylococcus aureus*, von Willebrand factor-binding protein

## Abstract

Von Willebrand factor-binding protein (vWbp), secreted by *Staphylococcus aureus* (*S. aureus*), can activate host prothrombin, convert fibrinogen to fibrin clots, induce blood clotting, and contribute to pathophysiology of *S. aureus*-related diseases, including infective endocarditis, staphylococcal sepsis and pneumonia. Therefore, vWbp is an promising drug target in the treatment of *S. aureus-*related infections. Here, we report that dryocrassin ABBA (ABBA), a natural compound derived from *Dryopteris crassirhizoma*, can significantly inhibit the coagulase activity of vWbp *in vitro* by directly interacting with vWbp without killing the bacteria or inhibiting the expression of the vWbp. Using molecular dynamics simulations, we demonstrate that ABBA binds to the “central cavity” in the elbow of vWbp by interacting with Arg-70, His-71, Ala-72, Gly-73, Tyr-74, Glu-75, Tyr-83, and Gln-87 in vWbp, thus interfering with the binding of vWbp to prothrombin. Furthermore, *in vivo* studies demonstrated that ABBA can attenuate injury and inflammation of mouse lung tissues caused by *S. aureus* and increase survival of mice. Together these findings indicate that ABBA is a promising lead drug for the treatment of *S. aureus*-related infections. This is the first report of potential inhibitor which inhibit the coagulase activity of vWbp by directly interacting with vWbp.

## Introduction

*Staphylococcus aureus* is one of the most common hospital-acquired pathogens, constituting approximately 20% of all hospital-acquired pathogens (Mertz et al., 2009; Tong et al., 2015). *S. aureus* causes a variety of diseases, such as skin infections and infections of the respiratory tract (Moise-Broder et al., 2004; Creech et al., 2015). Lung infections develop frequently in the hospital with high morbidity and mortality (Otto, 2014). The mortality rates of *S. aureus* community-acquired pneumonia was reported to be as high as 60% (David and Daum, 2010). Treatment of these infections is complicated because 40% of *S. aureus* isolates from patients with pneumonia are methicillin-resistant *S. aureus* (MRSA; Zhou et al., 2018). The development and spread of MRSA has become a growing challenge. Exploring new antimicrobial strategies has become an urgent problem to be solved.

There are almost 40 secreted virulence factors known to be associated with *S. aureus* infection (Diep et al., 2006). Coagulase is one of the important virulence factors. In previous research, coagulase has been indicated to facilitate the development of blood-borne staphylococcal pneumonia (Sawai et al., 1997). *S. aureus* secretes two coagulases, namely, staphylocoagulase (Coa; Friedrich et al., 2003) and von Willebrand factor-binding protein (vWbp; Bjerketorp et al., 2004). VWbp shares sequence homology with Coa and has a similar ability to bind to and activate prothrombin to form the staphylothrombin complex, thus bypassing the coagulation cascade that converts fibrinogen to fibrin and promoting the clotting of plasma (Cheng et al., 2010). VWbp is not essential for the growth of *S. aureus*, therefore, inhibition of vWbp hardly increases bacteria survival pressure and reduces the possibility of development of resistance. The search for vWbp inhibitors is of great significance in the treatment of *S. aureus* infections.

Only a few inhibitors of *S. aureus* coagulase have been reported. Yanagihara et al. (2006) found that a 21-bp siRNA that they designed and synthesized could inhibit the activity of *S. aureus* coagulase. However, the inhibitory effect of the siRNA was only approximately 40% of that of *S. aureus* mutant strain lacking coagulase (Yanagihara et al., 2006). *S. aureus* coagulase can directly activate prothrombin, bypassing the coagulation cascade that converts fibrinogen into fibrin. Therefore, anticoagulants such as low-molecular-weight heparin have no effect on the activity of coagulase in *S. aureus*. Similarly, calcium-chelating inhibitors, such as ethylenediaminetetraacetic acid (EDTA) and sodium citrate, which are usually used to inhibit *in vitro* blood coagulation, cannot inhibit the coagulation reaction induced by *S. aureus* (Peetermans et al., 2015), which is due to the combination of coagulase with the prothrombin binding region, which allows the exosite I of thrombin to be closed (Friedrich et al., 2003). As of 2010, some direct thrombin inhibitors have been found, such as dabigatran (Vanassche et al., 2010) and argatroban (Hijikata-Okunomiya and Kataoka, 2003), which can inhibit the activity of coagulase. Dabigatran can reduce fibrin formation on polyurethane catheters and can release renal abscesses (Vanassche et al., 2013). Dabigatran is commonly used to prevent stroke in patients with atrial fibrillation (Sander, 2017) and for the prevention and treatment of venous thromboembolism (Eriksson et al., 2007). Argatroban strongly inhibits the *S. aureus*-induced plasma clotting, the amidase activity of staphylothrombin and the fibrinogen-clotting activity of staphylothrombin (Hijikata-Okunomiya and Kataoka, 2003). Argatroban is often used as an anticoagulant for the treatment of heparin-induced thrombocytopenia (Hijikata-Okunomiya and Okamoto, 1992). However, few drugs so far have been reported for the treatment of *S. aureus*-induced diseases via direct inhibition of coagulase activity.

We have screened anti-vWbp molecules from 200 natural compounds via a blood-coagulation assay. We found that dryocrassin ABBA (ABBA) had relatively high inhibitory activity toward vWbp. ABBA (Figure 2A) is the tetrameric phlorophenone component derived from *Dryopteris crassirhizoma* (Kapadia et al., 1996). This compound suppresses the immunostimulatory function of dendritic cells and can prolong skin allograft survival (Fu et al., 2014). ABBA can also protect mice against the avian influenza virus H5N1 by inhibiting inflammation and reducing viral loads (Ou et al., 2015). In this study, the effect of ABBA on vWbp of *S. aureus* was investigated, and the potential therapeutic effect of ABBA on *S. aureus*-related pneumonia was assessed.

## Materials and Methods

### Bacterial Strains, Plasmids and Growth Conditions

The bacterial strains and plasmids used in this study are described in Table 1. *S. aureus* strains were cultured in brain-heart infusion (BHI) medium, which was supplemented with chloramphenicol (10 μg/ml) when required. *Escherichia coli* strains were cultured in Luria–Bertani (LB) medium, which was supplemented with ampicillin (100 μg/ml) when required.

**Table 1:** Strains and plasmids list.

Strain or plasmid	Relevant details	Source or reference
**Strains *S. aureus***		
Newman	Wild-type, srtA positive, hemolysis, coagulase positive	Newman
ΔvWbp	Newman harbors dCas9 and sgRNA-vWbp	Dong et al., 2017
***E. coli***		
DH5α	supE44 ΔlacU169 (Φ80 lacZDM15)	Invitrogen
	hsdR17 recA1 endA1 gyrA96 thi-1 relA1	
BL21	F^-^ *ompT hsdS (rB- mB-) gal dcm* (DE3)	Invitrogen
**Plasmids**		
pET15b	Expression vector	Amersham
vWbp-pET15b	pET15b with vWbp gene	This study

*S. aureus, Staphylococcus aureus, E. coli, Escherichia coli.*

### Preparation of Recombinant vWbp

The full-length coding sequence of mature vWbp was amplified by PCR from genome of *S. aureus* Newman using the primers 5′-GAACTCGAGGCATTATGTGTATCACAAATTTGGG-3′ (for ward) and 5′-GAAGGATCCGCAGCCATGCATTAATTATTTG CC-3′ (reverse). The PCR product was inserted into the XholI and the BamHI restriction sites of the pET15b vector, yielding pET15b-vWbp. For the overexpression of the vWbp, pET15b-vWbp was transformed into *E. coli* BL21 (DE3). Production of recombinant vWbp protein was induced with 0.5 mM IPTG after 12 h. Following induction, the cells were centrifuged at 4000 r/min for 30 min, suspended in 1 × column buffer (0.1 M Tris–HCl (pH 7.5), 0.5 M NaCl) and lysed with an ultrasonic disrupter. Lysates were centrifuged at 12000 r/min for 1 h, and the supernatant was subjected to Ni-NTA affinity chromatography. The column was washed with column buffer containing 40 mM imidazole, and the recombinant His-tagged vWbp protein was eluted with 500 mM imidazole and stored at –80°C.

### Coagulation Assay

For the tube coagulation assay, follow the protocol, sterile NaCl (0.9%) was added to Silin bottle containing freeze-dried rabbit plasma powder (purchased from Qingdao Hope Bio-Technology Co., Ltd.) and shake slightly to dissolve completely. 10 μL of recombinant vWbp (50 μM) and 490 μL above solution was added to borosilicate glass tubes and mixed. The tubes were incubated at 37°C, and coagulation was monitored by laying the tube down on its side every 10 min.

The plate coagulation assay was performed as described by Hwang et al. (1989) with minor modification. Agarose solution (0.9%) containing 0.4% PEG 8000, 3 mg/ml bovine fibrinogen and 1% plasma was added into 60-mm plates. Twenty microlitres of recombinant protein at concentrations ranging from 10 to 0.625 mg/ml was added to small wells punched out in the plates. Coagulation areas were measured after the plates were incubated at 37°C for 12 h.

### Determination of Minimum Inhibitory Concentration (MIC) and Growth Curves

The MIC of ABBA against *S. aureus* was measured by broth microdilution (Macia et al., 2014). To plot the growth curves of *S. aureus*, 1 ml of overnight-cultured *S. aureus* was added to 100 ml of sterile BHI broth with or without appropriate concentrations of ABBA. The absorbance value at 600 nm (OD_600_) was measured every 30 min for 24 h using an Infinite^®^ F200 PRO instrument.

### Western Blot Analysis

Western blotting was performed as previously described (Dong et al., 2013). *S. aureus* was cultured with different concentrations of ABBA to an OD_600_ of 0.6–0.8. Supernatant samples were collected by centrifugation, separated by SDS-PAGE, and transferred to polyvinylidene membranes. The membranes were blocked with 5% BSA for 2 h at room temperature. A specific anti-vWbp primary antibody was added at a 1:5000 dilution, and the membranes were then incubated overnight at 4°C. Next day, after washes thrice with PBS containing 0.05% Tween-20 (PBS-T), HRP-conjugated secondary goat anti-rabbit antiserum was added at a 1:2000 dilution, and the immunoreactive bands were visualized by an ECL Western blot detection system (GE Healthcare, United Kingdom) followed by 2 h of incubation.

### Thermal Shift Assay

SYPRO orange (5000×) was diluted into the buffer solution (1:100) and mixed with an equal volume of vWbp. Four microliters of the above mixture and 2 μL of ABBA were mixed with 14 μL of buffer solution in PCR tubes. Thermal scanning (25–95°C at 1°C/min) was performed using a Bio-Rad iQ5 real-time PCR instrument, and fluorescence values were measured every 10 s. The melting curve was plotted, and the temperature of the derivative curve peak was the Tm value of the protein. A Tm shift greater than 2°C compared to the Tm of the untreated protein was considered to be statistically significant (Krishna et al., 2013).

### Drug Affinity Responsive Target Stability (DARTS)

One microliter of different concentrations of ABBA was mixed with 19 μL of recombinant protein in Eppendorf (EP) tubes; the blank control group was treated with dimethyl sulfoxide (DMSO) instead of ABBA. The tubes were incubated at room temperature for 10 min. Pronase E was prepared on ice and added into each tube. The blank control group was treated with TNC buffer. Tubes were placed at 4°C for 30 min. Finally, 3 μL of cold 20 × protease inhibitor solution was added into the tubes to stop the reaction. The results were analyzed by SDS-PAGE.

### Homology Modeling and Molecular Docking

The X-ray crystallographic structure of the Coa-thrombin complex (PDB ID:1NU7) was used as a template for building the homology model of vWbp with Discovery Studio 2017 (Accelrys Inc., San Diego, CA, United States). The structure of ABBA was obtained from the PubChem database (CAS number: 12777-70-7). The parameters were at their default setting in the docking tab, and the grid maps were constructed to be large enough to include the binding sites of vWbp as well as significant regions of the surrounding surface. After molecular docking, the conformation with the highest consensus scoring pose was selected as the most likely binding conformation.

### Molecular Dynamics (MD) Simulations

Molecular dynamics (MD) simulation was performed to simulate the interactions between vWbp and ABBA using Discovery Studio 2017. The system was centered in a cubic box of TIP3P water molecules (Jorgensen et al., 2001). The system was first minimized by 5000 steps of steepest and 5000 steps of conjugate gradient. Then, the system was heated to the target temperature of 300 K for a period of 20 ps in constant pressure, periodic boundary conditions (NPT). Then, the program was set to equilibrate the system by 5 ns of constant pressure and temperature (NPT) with a time step of 2 fs, which was followed by 6 ns of production simulation performed under the same conditions. A cut off of 14 Å was used for non-bonded interactions, and long-range electrostatic interactions were treated by means of the particle mesh Ewald (PME) method (Darden et al., 2001). The MD simulation results were analyzed using Discovery Studio 2017.

### Determination of Catheter Fibrin Deposition by Scanning Electron Microscopy

The previously described *in vitro* catheter infection model was used with some minor modifications (Vanassche et al., 2013). Sterile, polyurethane, triple-lumen, central venous catheters were cut into 2-mm fragments. These fragments were placed in a suspension of either wild-type *S. aureus* Newman or *S. aureus* Newman vWbp-knockout strains (ΔvWbp) at an OD_600_ of 1.0 and incubated on a shaking platform at 37°C for 30 min. After being rinsed with sterile NaCl (0.9%), the catheters were placed in 1 ml of fresh rabbit plasma spiked with fibrinogen containing heparin with or without ABBA. After 24 h, catheters were rinsed with sterile NaCl (0.9%) and fixed overnight. Following a 2-h post-fixation period in 2% OsO_4_, the samples were dehydrated with a series of ethanol concentrations. After overnight immersion in hexamethyldisilazane, the samples were coated with platina and visualized using a JEOL 7401F scanning electron microscope (JEOL Europe, Zaventem, Belgium) at 2.0 kV.

### Mouse Model of *S. aureus* Pneumonia

Mice were bred and maintained under specific-pathogen-free conditions. The animal experiments were approved by and conducted in accordance with the principles of the Basel Declaration and the guidelines of the Animal Care and Use Committee of Jilin University. Eight-week-old C57BL/6J mice were obtained from Liaoning Changsheng Biotechnology Co., Ltd.

Female mice were divided into three groups: Newman, Newman + ABBA and ΔvWbp. After being anesthetized by ether, the mice were infected intranasally with 30 μL of a 6 × 10^8^ colony-forming units (CFU) *S. aureus* suspension. After 2 h, infected mice were subcutaneously injected with 100 mg/kg of ABBA or the same volume of DMSO with a 12-h interval. For survival research, mice were observed closely for signs of impending death at 12, 24, 36, 48, 60, 72, 84, and 96 h, if they appeared moribund (hunched posture, inability to move, dirty fur, labored breathing and unresponsive to external stimuli), mice were euthanized by carbon dioxide inhalation and were considered non-survivors. The isobole exponents were determined using the isobologram equation. For determination of CFU counts, mice were euthanized by carbon dioxide inhalation at 24 h post-infection. Left lungs were excised, weighed and homogenized for bacterial CFU counting by the serial dilution and plating method. Left lungs were fixed in 4% formalin and submitted for histopathological sectioning and haematoxylin-eosin staining, and the samples were then visualized by light microscopy.

### Statistical Analysis

The experimental data were performed using GraphPad Prism 5.0 (GraphPad Software) and were assessed using independent Student’s *t*-test with SPSS 22.0 statistical software. *P* values < 0.05 were considered statistically significant.

## Results

### ABBA Inhibits *S. aureus* the Coagulase Activity of vWbp in Coagulation Tests

**FIGURE 1:**
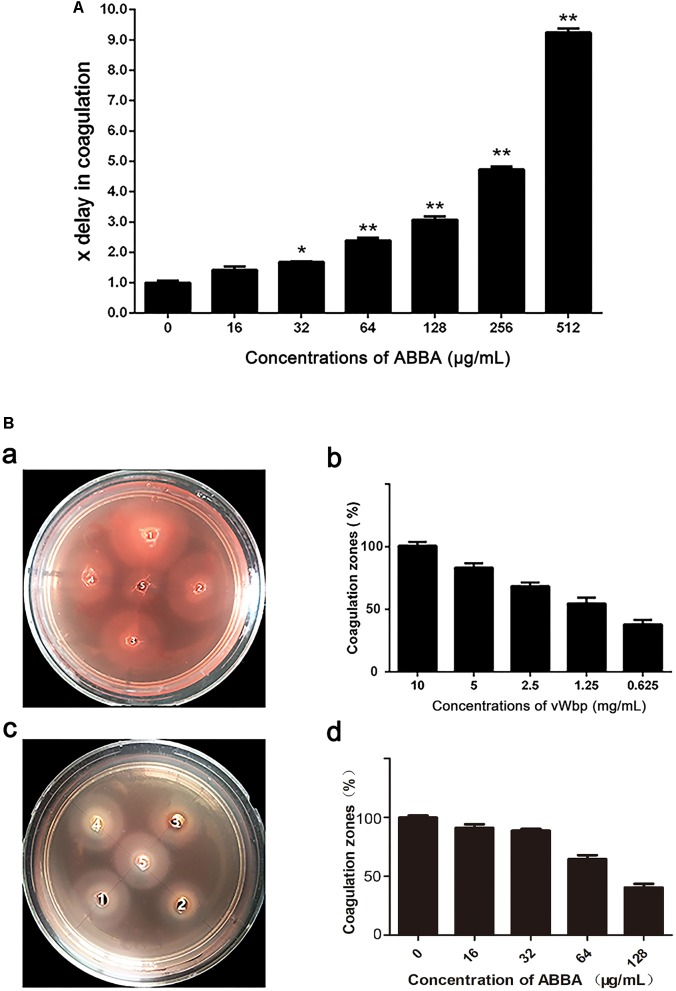
ABBA inhibits *S. aureus* vWbp Activity in tube and plate coagulation tests. **(A)** Recombinant vWbp was added to freeze-dried rabbit plasma mixed with different concentrations of ABBA. The plasma was incubated at 37°C, and coagulation was monitored by laying the tube down on its side every 10 min. **(B)** Agarose plate coagulation assay: 2-fold dilutions of vWbp (from well 1 to 5) were added to wells punched in plates containing rabbit plasma. Coagulation zones were measured after incubation at 37°C overnight **(a,b)**; 5 mg/ml vWbp was added to each well, and 128, 64, 32, and 16 μg/ml ABBA was added from well 2 to 5. Coagulation zones were measured after incubation at 37°C overnight **(c,d)**.

To determine the inhibitory effect of ABBA, the tube coagulation assay and the agarose plate assay were performed. The result of the tube coagulation assay showed the coagulation time increased in a dose-depend manner (Figure 1A), indicating that ABBA can inhibit the coagulase activity of vWbp. Serial dilutions of ABBA were added to tubes only conclude rabbit plasma. There were no coagulation in tubes after 24 h (Supplementary Figure 1A). In the agarose plate assay, fibrinogen is converted to fibrin to form turbid halos in the plates. The agarose plate assay was used to confirm the inhibitory activity of ABBA against vWbp. Figure 1Ba shows that the sizes of the coagulation zones increased with vWbp concentrations increasing from 0.625 to 10 mg/ml. The sizes of zones 2–5 were 83.24, 68.46, 54.68, and 38.02% of the size of well 1 (Figure 1Bb). The results indicate that vWbp converts fibrinogen to fibrin in a dose-dependent manner. Based on Figure 3A, 5 mg/ml vWbp was chosen for the subsequent experiment. Serial dilutions of ABBA (128, 64, 32, and 16 μg/ml) were added to wells 2–5 (Figure 1Bc). The sizes of zones of 2–5 were 30.39, 64.57, 88.67, and 92.02% of the size of well 1 (Figure 1Bd); 128 and 64 μg/ml of ABBA could inhibit the coagulase activity of vWbp significantly. Serial dilutions of ABBA were added to wells only conclude protein buffer. There were no turbid halos in the plates after 12 h (Supplementary Figure 1B).

### ABBA Does Not Perturb the Growth State of *S. aureus*

**FIGURE 2:**
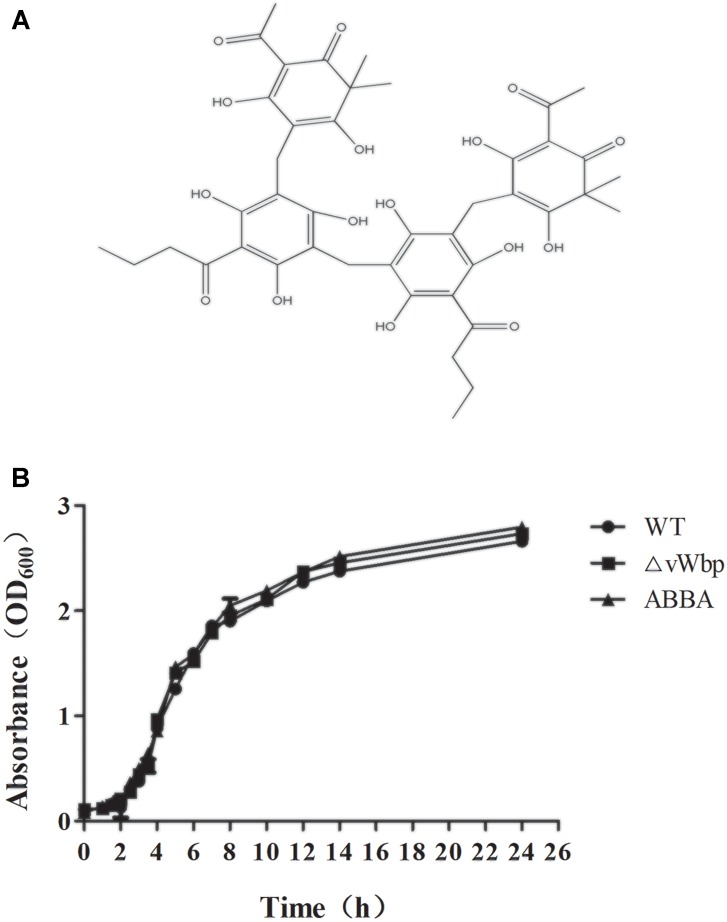
Structure of ABBA **(A)** and growth curves of *S. aureus* Newman with or without ABBA (512 μg/ml) and ΔvWbp **(B)**.

The MICs of ABBA on *S. aureus* Newman were determined to be greater than 1024 μg/ml via the broth microdilution method. The growth curves of *S. aureus* Newman with or without ABBA (512 μg/ml) did not differ significantly. The growth curve of the vWbp mutant also did not differ significantly from that of *S. aureus* Newman (Figure 2B). These results indicate that ABBA could be a potential small-molecule inhibitor of vWbp that is effective at a concentration far lower than its MIC.

### ABBA Has No Effect on the Expression of vWbp

**FIGURE 3:**
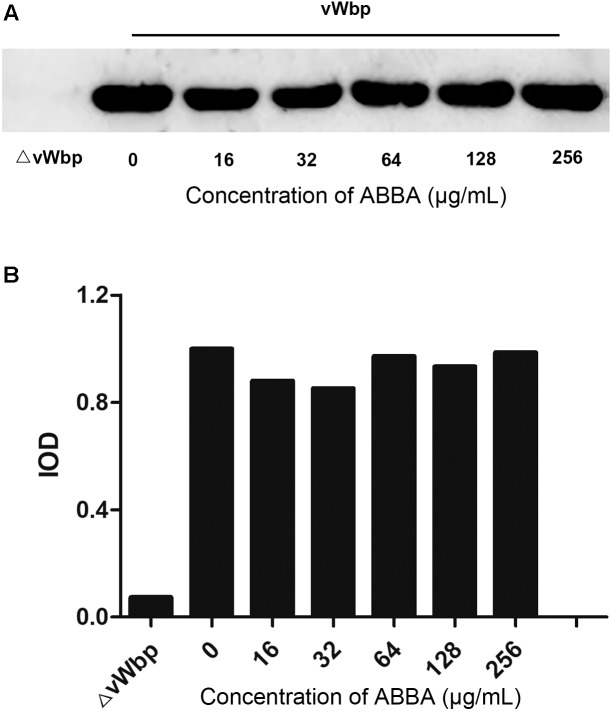
Western blot analysis of vWbp expression levels. **(A)** Western blotting was performed to detect the expression levels of vWbp incubated with 16, 32, 64, 128 or 256 μg/ml ABBA and ΔvWbp. **(B)** Analysis of gray levels of the protein bands.

To examine whether the inhibitory effect of ABBA on *S. aureus*-induced coagulation by affecting the expression of vWbp, we extracted whole cell proteins of *S. aureus* cultures treated with 16, 32, 64, 128, and 256 μg/ml ABBA. The vWbp expression levels were determined by Western blotting. The gray values of blot of the different treated samples were further analyzed for confirmation of the expression levels. The results revealed that the expression levels were similar at different concentrations of ABBA (Figures 3A,B). The result shows that ABBA did not affect vWbp expression.

### ABBA Can Improve the Thermal Stability of vWbp

**FIGURE 4:**
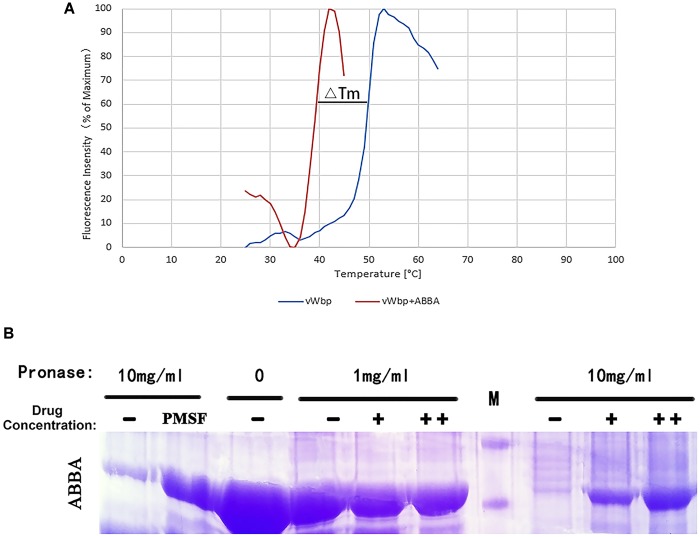
Fluorescence-based thermal shift assay results and representative DARTS results for pronase E-digested vWbp. **(A)** To identify whether ABBA can improve the thermal stability of vWbp, vWbp was subjected to a thermal shift assay in the presence of 64 μg/ml ABBA (red curves) or buffer only (blue curves). **(B)** SDS-PAGE bands from left to right: 1, negative control, vWbp + 10 mg/ml pronase E; 2, positive control, vWbp +10 mg/ml pronase E + PMSF (inhibitor of pronase E); 3, vWbp; 4, vWbp + 1 mg/ml pronase E; 5, vWbp + 1 mg/ml pronase E + 64 μg/ml ABBA; 6, vWbp + 1 mg/ml pronase E + 128 μg/ml ABBA; 7, marker; 8, vWbp + 10 mg/ml pronase E; 9, vWbp + 10 mg/ml pronase E + 64 μg/ml ABBA; 10, vWbp + 10 mg/ml pronase E + 128 μg/ml ABBA.

Binding with low-molecular-weight ligands can increase the thermal stability of a protein, as described by Koshland et al. (1958). Proteins unfold when heated. SYPRO orange binds to hydrophobic surfaces non-specifically, leading to an increase in fluorescence intensity. The fluorescence intensity starts to decrease after maximum binding is achieved. In general, a curve shift greater than 2°C is considered a statistically significant change in measured Tm when determining ligand-binding affinity. In Figure 4A, the blue curve is the melting curve of vWbp, and the red curve is the melting curve of vWbp incubated with ABBA (64 μg/ml). Comparison of the blue and red curves shows that Tm value changed from 39 to 50°C. This 11°C shift is statistically significant. The results showed that ABBA can improve the thermal stability of vWbp, indicating that ABBA can directly interact with vWbp.

### ABBA Affects the Digestion of vWbp by Pronase E

Binding of drugs has been proposed to stabilize target proteins, either globally or locally. This stabilization can occur due to a specific conformation or simply via the masking of protease recognition sites, thereby reducing the protease sensitivity of the target protein. DARTS is a general methodology for identifying and studying protein-ligand interactions. The principle of this technique is that when a small molecule compound binds to a protein, the interaction make the protein becoming protease resistant by stabilizing the protein structure (Lomenick et al., 2009). DARTS was performed to further identify the direct binding of ABBA to the vWbp. As shown in Figure 4B, when the concentration of pronase E was 10 mg/ml, vWbp was digested completely. Addition of ABBA protected vWbp from digestion, and the digestibility was associated with the concentration of ABBA. The results demonstrate that ABBA can directly interact with vWbp and stabilize vWbp. Furthermore, with increasing drug concentration, the drug molecules binding to the vWbp protein also increased.

### Determination of the vWbp-ABBA Binding Mechanism

**FIGURE 5:**
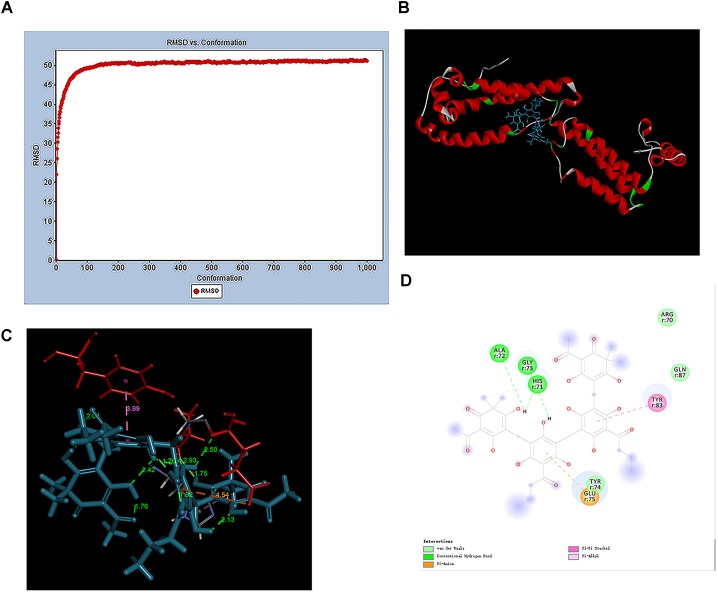
General view of the vWbp-ABBA complex based on MD simulation. **(A)** RMSD plot for the vWbp-ABBA complex during a 20-ns MD simulation. **(B)** A binding model of the vWbp-ABBA complex was prepared by using Discovery Studio 2017. **(C)** Details of the binding model of the vWbp-ABBA complex. **(D)** A 2D diagram of the vWbp-ABBA complex was prepared by using Discovery Studio 2017.

Previous research has demonstrated the interaction between vWbp and ABBA. To investigate the mechanism of this interaction, the vWbp-ABBA complex was equilibrated after 20 ns of MD simulation. The root-mean-square deviation (RMSD) values of vWbp were plotted as a curve (Figure 5A). The results showed that the vWbp structures of all the systems were stabilized during the simulations. The theoretical binding mode of ABBA and vWbp is shown in Figure 5B. ABBA adopted a compact conformation, binding at the “central cavity” in the elbow of vWbp. Specifically, as shown in Figures 5C,D, the hydroxyl group at the 2-position in the diene of ABBA formed conventional hydrogen bonds with the residues His-71 and Ala-72. The hydroxyl group at the 2-position in the phenyl group of ABBA formed conventional hydrogen bonds with the residue Gly-73. The phenyl group of ABBA exhibited a pi-anion interaction with residue Glu-75. Another phenyl group of ABBA exhibited pi–pi stacking with residue Tyr-83. Moreover, the residues Arg-70, Tyr-74, and Gln-87 can form van der Waals interactions with ABBA. In brief, the above MD simulations provided a rational prediction of the interactions between ABBA and vWbp.

### ABBA Reduces Adherent Biomaterial Deposition on Catheters *in vitro*

**FIGURE 6:**
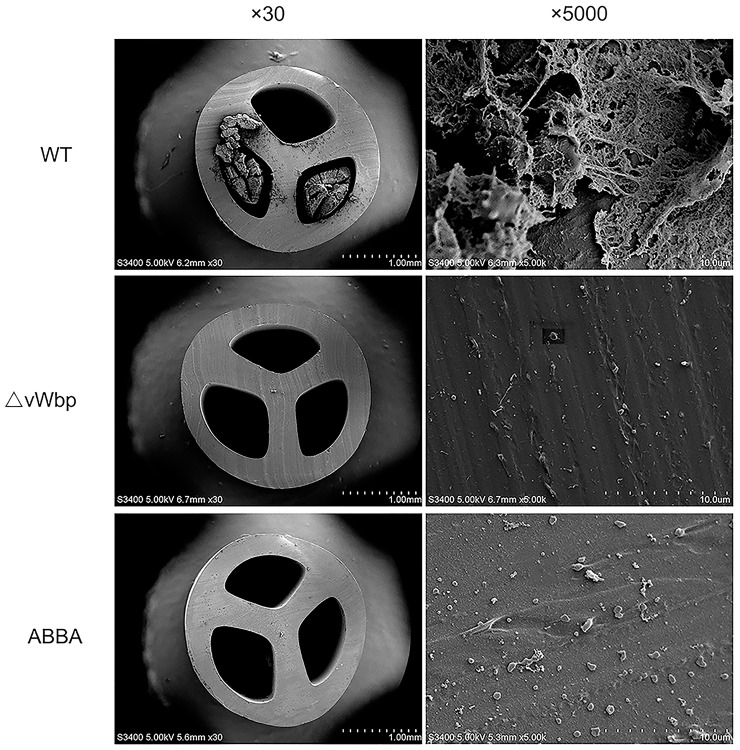
Scanning electron microscopy of catheters inoculated with *S. aureus* strains harboring or lacking the vWbp gene. Abundant adherent material was seen on the surfaces of catheters incubated in heparin-spiked plasma for 24 h (top row). Both deletion of vWbp (middle row) and inhibition by ABBA at a concentration of 128 μg/ml (bottom row) reduced the adherent material, leaving cocci on surface of catheter without surrounding matrix.

The results of scanning electron microscopy showed the presence of abundant adherent material on catheter fragments inoculated with *S. aureus* Newman WT as well as heparin-spiked plasma for 24 h (Figure 6, top row). The adherent biomaterial consisted of fibrin and dispersed cocci. The amount of adherent material was significantly reduced in the presence of ABBA (128 μg/ml). Only a small number of cocci were observed to be directly adhered to the catheter surface without any surrounding matrix (Figure 6, bottom row). Similarly, the ΔvWbp strain was unable to induce this rapid deposition of adherent biomaterial (Figure 6, middle row).

### ABBA Protects Mice From *S. aureus* Pneumonia

**FIGURE 7:**
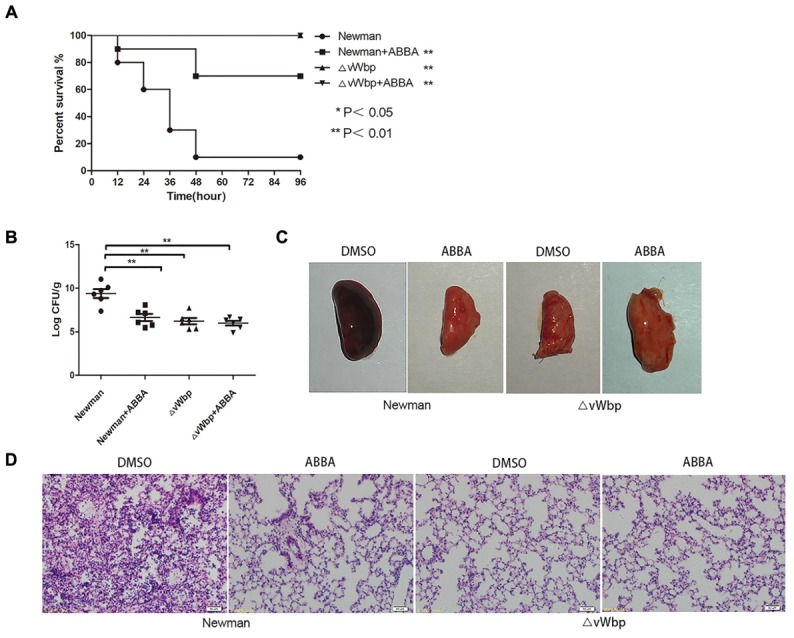
ABBA protects mice against *S. aureus* pneumonia. Mice were infected via intranasal administration with *S. aureus* Newman or *S. aureus* Newman ΔvWbp. **(A)** Survival was monitored at 12, 24, 36, 48, 60, 72, 84, and 96 h (*n* = 10). The asterisks indicate statistical significance versus the Newman-infected group using the log-rank test. **(B)** CFU counts in the lungs of infected mice 24 h post-infection. Gross pathological changes **(C)** and histopathology **(D)** of lung tissue infected with *S. aureus*, which was treated with either DMSO or ABBA (*n* = 6). ^∗∗^ indicates *P* < 0.01 compared with the Newman group and Newman ΔvWbp group.

Von Willebrand factor-binding protein has been reported to be an important virulence factor in mouse models of *S. aureus* pneumonia (Sawai et al., 1997). Based on the *in vitro* observation that ABBA can inhibit the blood coagulation activity of the vWbp, a mouse model of *S. aureus* pneumonia was established to investigate the *in vivo* protective effects of ABBA. Mice were infected with 30 μL of *S. aureus* Newman (2 × 10^8^ CFU/10 μL) by intranasal administration. Then, the mice were subcutaneously injected with either DMSO or 100 mg/kg ABBA every 12 h. The mice infected with ΔvWbp strain were used as negative controls. The survival was monitored every 12 h. And the result shows that mice treated with ABBA were resulted a significant increase in survival (*P* < 0.01) than mice treated with DMSO (Figure 7A). 70% of mice in ABBA group were survived over the 96 h experiment. And there were no death in ΔvWbp group and ΔvWbp + ABBA group.

The numbers of CFU in the lungs were counted to evaluate the effects of ABBA in the treatment of pneumonia. As shown in Figure 7B, the CFU counts in the lungs infected mice treated with ABBA (6.62 ± 1.01 log CFU/g) were significantly lower than those in the lungs of mice treated with DMSO (9.38 ± 1.24 log CFU/g) (*P* < 0.01). As a comparison, the CFU counts in the lungs of mice infected with the ΔvWbp strain were also low (6.21 ± 0.36 log CFU/g). The CFU counts in lungs infected with the ΔvWbp strain and treated with ABBA (5.97 ± 0.65 log CFU/g) were also significantly lower than Newman group, which indicate that there were no off-target effects. In addition, the lung tissues of mice treated with ABBA were pink, soft and elastic, while those of mice treated with DMSO were dark red, swollen and inelastic (Figure 7C). Further histopathological research showed that more alveolar destruction, pulmonary hyperaemia and greater inflammatory cell infiltration occurred in infected mice treated with DMSO than in those treated with ABBA (Figure 7D). The results revealed that ABBA can reduce infection and prevent mice from contracting *S. aureus* pneumonia.

## Discussion

*Staphylococcus aureus* has become one of the most common pathogens and causes life-threating infections (Dantes et al., 2013). *S. aureus*-related pneumonia is one of the most common invasive infections. In nosocomial pneumonia infections, the rate of MRSA emergence was reported to be increasing, which has made the disease difficult to cure (Koomanachai et al., 2009). While this disease can be treated with vancomycin (first-choice drug), the rate of mortality remains high and the appearance of intermediate resistance limits the usefulness of this class of antibiotics (Walkey et al., 2011; Docobo-Perez et al., 2012). Anti-virulence strategies are aimed at reducing bacterial toxicity and eliminating bacteria through the host’s immune system, which decreases the development of drug resistance in bacteria. Therefore, the identification of inhibitors that target virulence factors is a promising approach for the treatment of diseases caused by *S. aureus* infection.

*Staphylococcus aureus* secretes two kinds of coagulases: Coa and vWbp. Prothrombin is conformationally activated by these two secreted proteins, and fibrinogen is converted to insoluble fibrin by staphylothrombin (Friedrich et al., 2003). Previous research has shown that coagulase can facilitate the development of blood-borne staphylococcal pneumonia (Sawai et al., 1997). In this study, we identified vWbp to be a key protein involved in staphylococcal pneumonia. As a virulence factor, the inhibition of vWbp did not affect the growth of *S. aureus*, resulting in a decrease in the development of resistance due to the absence of selective pressures, in contrast to traditional approaches.

Currently, two effective and commonly used small molecule inhibitors, dabigatran and agatroban, are used to protect the body against *S. aureus* infections by inhibiting staphylothrombin. However, notably, these two inhibitors can inhibit not only staphylothrombin but also thrombin produced by the physiological coagulation cascade (Peetermans et al., 2015), which can easily lead to adverse reactions, such as bleeding. Therefore, the development of inhibitors that are unable to affect the physiological thrombin activity of the host, but can specifically inhibit the coagulase of *S. aureus*, has high clinical value. At present, inhibitor that directly acts on vWbp has not been reported.

We screened anti-vWbp molecules from 200 natural compounds via a blood coagulation assay. ABBA was found to have significant inhibitory activity against vWbp at a low concentration. Previous studies have reported that ABBA can prolong skin allografts (Fu et al., 2014) and protect mice against the influenza virus H5N1 (Ou et al., 2015). In this study, the DARTS result revealed that ABBA protects vWbp from digestion by pronase E. In addition, the result of the thermal shift assay proved that ABBA stabilized vWbp when heated. These two experiments provided substantial evidence of the direct interaction between ABBA and vWbp.

A study by Zimmerli et al. (2004) demonstrated that the capacity of staphylococci to embed in an extracellular matrix played an important role in chronic and persistent infections such as pneumonia, endocarditis, and foreign-body-associated infections. We observed that ABBA reduced the coating of catheter fragments with a dense fibrin matrix, only a small number of cocci remained. However, large amounts of fibrin matrix could be seen on the catheters incubated with *S. aureus* Newman, suggesting that ABBA destroyed the protective microenvironment for *S. aureus* growth on the catheter surface. We also evaluated the *in vivo* effects of ABBA in a C57BL/6J mouse model. The results indicate that ABBA can reduce the bacterial load in lungs and alleviate the pulmonary infections induced by *S. aureus* Newman.

In this study, we also modeled the binding mechanism of ABBA with vWbp by MD simulation. The results indicated that ABBA binds at the elbow region of vWbp. Importantly, the Arg-70, His-71, Ala-72, Gly-73, Tyr-74, Glu-75, Tyr-83, and Gln-87 residues play key roles in the binding of ABBA to vWbp. ABBA directly binds to the “central cavity” in the elbow of vWbp, thus interfering with the binding of vWbp with prothrombin. Via inhibition of vWbp, fibrin production was decreased.

In conclusion, our findings demonstrate that ABBA can inhibit the blood coagulation activity of vWbp by direct interaction, offering protection to mice from *S. aureus* pneumonia, improving lung function, causing bacterial clearance and increasing survival. This is the first report of potential inhibitor which inhibit the coagulase activity of vWbp by directly interacting with vWbp. Therefore, ABBA can be considered to be a promising candidate for the treatment of *S. aureus* pneumonia.

## Author Contributions

DW, LW, and TW designed the study. BL, DM, and PY performed the *in vitro* experiments. XL, LZ, JC, and DX performed the *in vivo* experiments. BL and YJ wrote the manuscript. HX and QG edited and modified the manuscript.

## Conflict of Interest Statement

The authors declare that the research was conducted in the absence of any commercial or financial relationships that could be construed as a potential conflict of interest.

## Supplementary Material

FIGURE S1 The infection of ABBA on only plasma in tube and plate coagulation tests. **(A)** Serial dilutions of ABBA were added to tubes only conclude freeze-dried rabbit plasma. The tubes were incubated at 37°C, and coagulations were monitored by laying the tubes down on sides every 10 min. **(B)** Serial dilutions of ABBA (from well 1 to 4) were added to wells punched in plates only conclude protein buffer. Coagulation zones were measured after incubation at 37°C overnight.
