# Supplementary figures and images for: An Inhibitory Effect of Dryocrassin ABBA on Staphylococcus aureus vWbp That Protects Mice From Pneumonia
Source: Front Microbiol. 2019 Jan 23;10:7. doi: 10.3389/fmicb.2019.00007 (PMC6351477; doi:10.3389/fmicb.2019.00007)

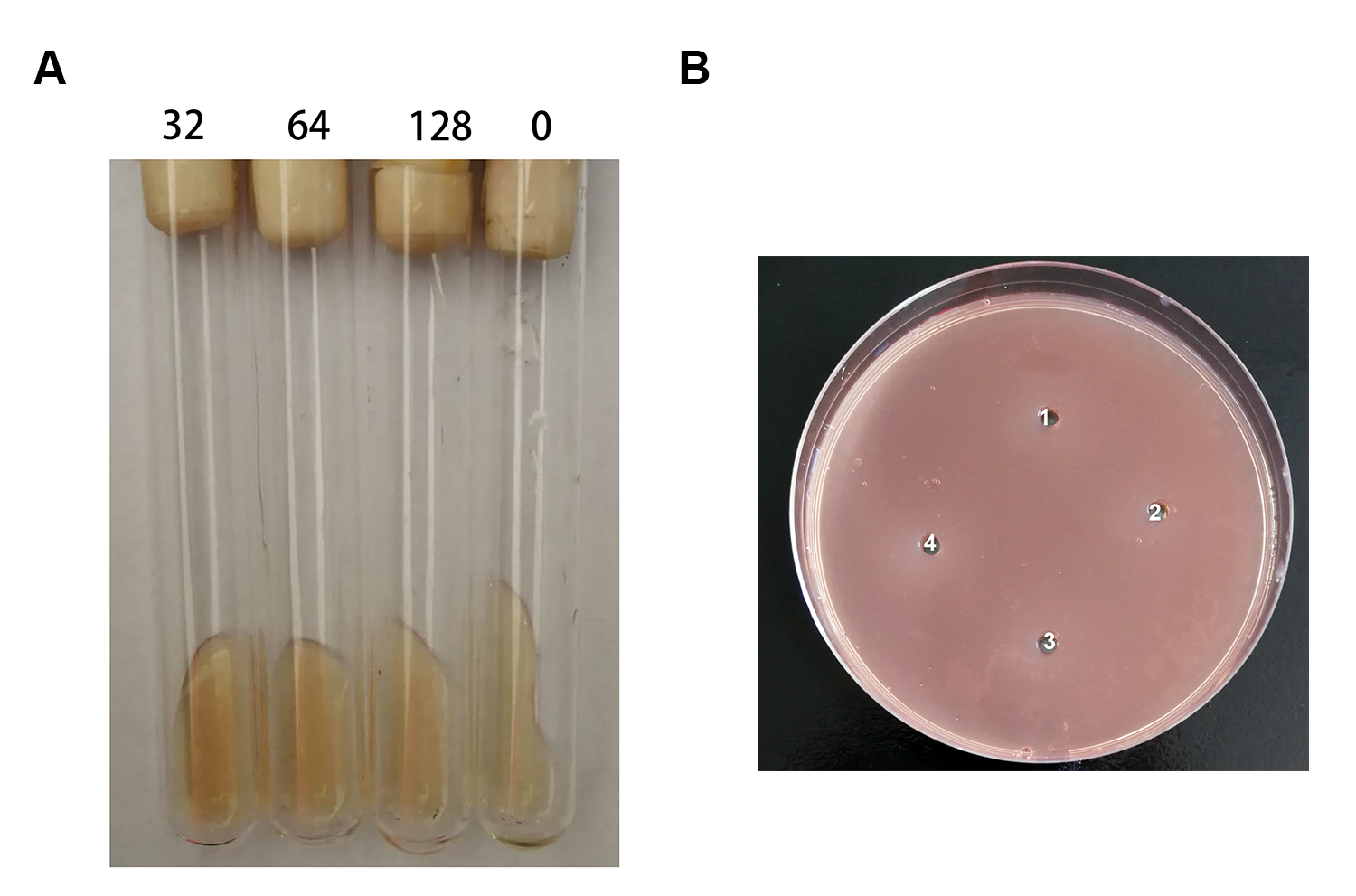

Supplement: FIGURE S1 — The infection of ABBA on only plasma in tube and plate coagulation tests. (A) Serial dilutions of ABBA were added to tubes only conclude freeze-dried rabbit plasma. The tubes were incubated at 37°C, and coagulations were monitored by laying the tubes down on sides every 10 min. (B) Serial dilutions of ABBA (from well 1 to 4) were added to wells punched in plates only conclude protein buffer. Coagulation zones were measured after incubation at 37°C overnight. [file Image_1.tif]
